# State of Ireland's mental health: findings from a nationally representative survey

**DOI:** 10.1017/S2045796022000312

**Published:** 2022-07-01

**Authors:** Philip Hyland, Frédérique Vallières, Mark Shevlin, Richard P. Bentall, Sarah Butter, Todd K. Hartman, Thanos Karatzias, Anton P. Martinez, Orla McBride, Jamie Murphy, Robert Fox

**Affiliations:** 1 Department of Psychology, Maynooth University, Kildare, Ireland; 2 Trinity Centre for Global Health, University of Dublin, Trinity College, Dublin, Ireland; 3 School of Psychology, Ulster University, Derry, Northern Ireland; 4 Department of Psychology, The University of Sheffield, England; 5 Bamford Centre for Mental Health and Wellbeing, Ulster University, Derry, Northern Ireland; 6 Department of Social Statistics, The University of Manchester, Manchester, England; 7 School of Health & Social Care, Edinburgh Napier University, Edinburgh, Scotland; 8 School of Business, National College of Ireland, Dublin, Ireland

**Keywords:** Comorbidity, Ireland, mental health, prevalence, suicide

## Abstract

**Aims:**

Current information about the prevalence of various mental health disorders in the general adult population of the Republic of Ireland is lacking. In this study, we examined the prevalence of 12 common mental disorders, the proportion of adults who screened positive for any disorder, the sociodemographic factors associated with meeting criteria for a disorder and the associations between each disorder and history of attempted suicide.

**Methods:**

A non-probability nationally representative sample (*N* = 1110) of adults living in Ireland completed self-report measures of 12 mental health disorders. Effect sizes were calculated using odds ratios from logistic regression models, and population attributable risk fractions (PAFs) were estimated to quantify the associations between each disorder and attempted suicide.

**Results:**

Prevalence rates ranged from 15.0% (insomnia disorder) to 1.7% (histrionic personality disorder). Overall, 42.5% of the sample met criteria for a mental health disorder, and 11.1% had a lifetime history of attempted suicide. Younger age, being a shift worker and trauma exposure were independently associated with a higher likelihood of having a mental health disorder, while being in university was associated with a lower likelihood of having a disorder. ICD-11 complex posttraumatic stress disorder, borderline personality disorder and insomnia disorder had the highest PAFs for attempted suicide.

**Conclusions:**

Mental health disorder prevalence in Ireland is relatively high compared to international estimates. The findings are discussed in relation to important mental health policy implications.

Mental health disorders are common, costly and crippling. Together, they remain one of the leading causes of ‘years lost to disability’ worldwide, with an estimated 125.3 million years of productive life lost in 2019 alone (Global Burden of Disease (GBD) 2019 Mental Disorders Collaborators, 2022). The global economic cost of mental health disorders is US$2.5 trillion and is projected to rise to US$6 trillion by 2030 (Trautmann *et al*., 2016). Moreover, mental health disorders increase the risk of other adverse health outcomes (i.e. morbidity), are associated with a marked decrease in life expectancy (Chang *et al*., 2011) and contribute to the over 800 000 suicide deaths recorded annually (GBD 2017 Causes of Death Collaborators, 2018; GBD, 2022). Psychological autopsy studies suggest that 90% of people who died by suicide had a mental health disorder at the time of their death (Brådvik, 2018).

Despite the devastating human and economic impact of mental disorders, it is difficult to ascertain what proportion of the general population has a mental health disorder at any given time. Reasons for this include the limited number of disorders that can reasonably be assessed in any given study, the use of different assessment methods (e.g. structured clinical interviews or self-report measures) that can produce discrepant prevalence estimates (Hoffman *et al*., 2011) and varying social norms within and across nations regarding willingness to disclose personal information about mental health (Krendl and Pescosolido, 2020). That said, progress has been made in recent years.

Data collected from 17 countries as part of the World Health Organization's (WHO) World Mental Health Survey found that the proportion of the general adult population meeting criteria for any mental health disorder at any given time ranged from 12% in Nigeria to 47% in the USA, with an inter-quartile range of 18–36% (Kessler *et al*., 2007). Reliable estimates also come from prospective birth cohort studies. New Zealand's Dunedin Birth Cohort Study (*N* = 1037), for example, assessed 11 disorders using diagnostic interviews on six occasions when participants were aged between 18 and 45 years. At each assessment, between 44% (age 45) and 50% (at age 18) of people met criteria for at least one mental health disorder, and 86% experienced a disorder by age 45 (Caspi *et al*., 2020). Similar figures have been observed in other birth cohort studies (Schaefer *et al*., 2017). Findings from these studies suggest that not only do nearly half of the adult general population meet requirements for a mental health disorder at any point in time, but that there are high levels of comorbidity between the different mental health disorders. In one salient example, Plana-Ripoll *et al*. (2019) analysed Danish registry data from six million people over a 16-year period and found that having any mental health disorder increased the probability of subsequently being diagnosed with every other mental health disorder.

Consistent with the Lancet Commission on global mental health and sustainable development's call for more investment in mental health services (Patel *et al*., 2018), the Government of Ireland (2020) announced a new national mental health policy identifying the ‘*expansion of mental health services to address the spectrum of conditions and needs*’ as a priority for the next decade. Determining the appropriate level of mental health services required to meet the nation's mental health need requires an accurate understanding of the occurrence and co-occurrence of mental health disorders in the population. Several studies in Ireland suggest that the prevalence of major depressive disorder (MDD) is 6%, generalised anxiety disorder (GAD) is 5%, ICD-11 posttraumatic stress disorder (PTSD) is 5%, ICD-11 complex PTSD (CPTSD) is 7.7% and lifetime history of attempted suicide stands at 11% (Barry *et al*., 2009; Hyland *et al*., 2021a, 2022). Additionally, the WHO's Global Status Report on Alcohol and Health estimated the prevalence of alcohol use disorder (AUD) in Ireland at 8.5% (WHO, 2018). To date, however, no single study has investigated the prevalence of a wide array of mental health disorders simultaneously in the Irish population meaning it has been impossible to ascertain what proportion of people meet requirements for a mental health disorder.

Therefore, this study was performed to provide the first comprehensive assessment of the occurrence and co-occurrence of multiple mental health disorders in the adult population of Ireland. Three major study objectives were formulated. The first was to determine (a) the prevalence rates of 12 mental health disorders, (b) the proportion of adults meeting criteria for any disorder and (c) the correlations among these disorders. The second objective was to identify sociodemographic characteristics associated with meeting criteria for any mental health disorder. Finally, the third objective was to determine (a) the association between meeting criteria for any mental health disorder and attempted suicide, (b) which mental health disorders were uniquely associated with attempted suicide and (c) the proportion of attempted suicides uniquely attributable to specific mental health disorders.

## Methods

### Participants and procedures

This study was based on data collected at wave 5 (*N* = 1100) of the Irish arm of the COVID-19 Psychological Research Consortium Study; a multinational, longitudinal, Internet-based research project assessing the population's psychological and social adjustments to the pandemic (McBride *et al*., 2021). Data were collected between 19 March and 9 April 2021 by the survey company Qualtrics. Participants were recruited from actively managed, double-opt-in research panels via email, SMS or in-app notifications, and were selected using quota sampling methods to produce a sample representative of the entire adult population of the Republic of Ireland in terms of sex, age and geographical distribution, as per the most recent Irish census (Central Statistics Office, 2016). Inclusion criteria required that respondents be aged 18 years or older, reside in the Republic of Ireland, and be capable of completing the survey in English. Participants were remunerated by Qualtrics, and informed consent was obtained from all participants. Ethical approval was granted by the Social Research Ethics Committee at Maynooth University [SRESC-2020-2402202]. Table 1 provides sociodemographic details of the sample.

**Table 1.** Sociodemographic characteristics of the sample (*N* = 1100)

	*N*	%
Sex		
Male	533	48.0
Female	574	51.7
Transgender, prefer not to say, or other	3	0.3
Age		
18–24	124	11.2
25–34	214	19.3
35–44	236	21.3
45–54	180	16.2
55+	356	32.1
Born in Ireland		
Yes	847	76.3
No	263	23.7
Ethnicity		
Irish	902	81.3
Non-Irish	208	18.7
Area of residence		
City	276	24.9
Suburb	236	21.3
Town	311	28.0
Rural	287	25.9
Highest education achievement		
Did not finish secondary school	67	6.0%
Finished secondary school	249	22.4%
Undergraduate degree	300	27.0
Postgraduate degree	213	19.2
Other qualification (diploma, technical degree)	281	25.3
Religion		
Atheist or agnostic	199	17.9
Theist	911	82.1
Relationship status		
In a committed relationship	742	66.8
Single	278	25.0
Separated/divorced	63	5.7
Widowed	27	2.4
Living alone		
No	947	85.3
Yes	163	14.7
Do you have children?		
No	437	39.4
Yes, living with me	467	42.1
Yes, not living with me	206	18.6
Employment status		
Full-time employed	505	45.5
Part-time employed	164	14.8
Temporary working arrangement	23	2.1
Retired	172	15.5
Unemployed	122	11.0
Temporarily unemployed due to COVID-19	66	5.9
Full-time student	58	5.2
Income level		
€0–19 999	337	30.4
€20 000–29 999	240	21.6
€30 000–39 999	207	18.6
€40 000–49 999	150	13.5
€50 000 or more	176	15.9
Shift worker (working between 12am and 7am)		
No	958	86.3
Yes	152	13.7
Physical health condition (diabetes, cancer, heart disease, lung disease)		
No	828	74.6
Yes	282	25.4
Trauma exposed		
No	332	29.9
Yes	778	70.1

Empirical analyses show that the current sample is highly representative of the known, census-derived population parameters on each of the quota variables with sample proportions all falling within ~1% of the known population parameters (Spikol *et al*., 2021). Moreover, the sample was also reasonably representative of the population on several non-quota variables such as ethnicity, religious affiliation, educational achievement and employment status. *A priori* power analyses were conducted to determine the optimal sample size for identifying mental health disorders in the general population with a prevalence of 5% (see Barry *et al*., 2009; Hyland *et al*., 2021a). A sample size of 1842 was necessary to detect a disorder with a 5% prevalence with a precision of 1% and 95% confidence. However, Qualtrics was only able to guarantee 1000 participants so the target sample size was set at 1000 which, holding all other parameters in the sample size calculation equal, resulted in a precision of 1.35%.

### Measures

Twelve mental health disorders were assessed using self-report measures, selected for their well-established guidelines for identifying participants likely to meet criteria for a mental health disorder (Farber *et al*., 2020). Since self-report measures have been found to produce higher prevalence estimates than clinician-administered diagnostic interviews (Hoffman *et al*., 2011; Levis *et al*., 2020), the most stringent cut-off scores associated with a given measure were used for disorder identification.

#### MDD and GAD

MDD and GAD were measured using the *Patient Health Questionnaire-9* (PHQ-9; Kroenke *et al*., 2001) and the *Generalised Anxiety Disorder 7-item Scale* (GAD-7; Spitzer *et al*., 2006), respectively. These measures ask participants to indicate how often they have been bothered by each symptom over the last 2 weeks using a four-point Likert scale ranging from 0 (‘Not at all’) to 3 (‘Nearly every day’). Total scores range from 0 to 27 and 0 to 21 on the two measures, and scores ⩾10 and ⩾15 have been recommended for identifying participants with probable diagnostic status (Levis *et al*., 2020). The most stringent cut-off of ⩾15 was used to identify cases of MDD and GAD. These measures have been shown to produce reliable and valid scores in general population surveys (Hinz *et al*., 2017; Shin *et al*., 2020). The PHQ-9 (*α* = 0.92) and GAD-7 (*α* = 0.94) had excellent internal reliability in this sample.

#### ICD-11 PTSD and CPTSD

The *International Trauma Questionnaire* (ITQ; Cloitre *et al*., 2018) captures all diagnostic criteria for PTSD and CPTSD according to the ICD-11 diagnostic rules. Participants were first asked to indicate if they were ever exposed to any of 16 traumatic life events taken from the *International Trauma Exposure Measure* (Hyland *et al*., 2021b), and, in the case of multiple trauma exposures, to nominate their worst traumatic event. Participants were subsequently asked to answer all symptom measures in relation to this worst traumatic event. In total, 70.1% of people had experienced at least one traumatic event. There are six items measuring PTSD symptoms of re-experiencing in the here and now, avoidance and sense of current threat, and participants indicate how bothered they have been by these symptoms over the past month. Another six items measure the ‘disturbance in self-organisation’ (DSO) symptoms of affective dysregulation, negative self-concept and disturbed relationships, and these are answered in terms of typical reactions. Both sets of symptoms are followed by measures of functional impairment across different domains of life. All items are answered using a five-point Likert scale ranging from 0 (‘Not at all’) to 4 (‘Extremely’), where a symptom is considered present based on a response of ⩾2 (‘Moderately’). Diagnostic requirements for PTSD include trauma exposure, one symptom present from each PTSD cluster and presence of functional impairment associated with these symptoms. Diagnosis of CPTSD requires that all PTSD criteria are met, as well at least one symptom present from each DSO cluster and evidence of functional impairment associated with these symptoms. The ICD-11 diagnostic rules permit a diagnosis of PTSD or CPTSD, but not both. Thus, if a person meets criteria for CPTSD, they cannot also receive a PTSD diagnosis. The psychometric properties of the ITQ are well supported (Redican *et al*., 2021), and the internal reliability of the scale scores in this sample was excellent (*α* = 0.95).

#### Insomnia disorder

The *Sleep Condition Indicator* (SCI; Espie *et al*., 2014) is an eight-item measure developed to screen for DSM-5 insomnia disorder. Participants report on different types of sleep problems, sleep dissatisfaction and consequences of poor sleep experienced during the last month. All items use a five-point Likert scale (0–4) with possible total scores ranging from 0 to 32. A given symptom is present based on a score of ⩾2 on the Likert scale, and the criteria for DSM-5 insomnia disorder are met when there is evidence of: (a) difficulty initiating or maintaining sleep, (b) significant distress, (c) frequency of problems and (d) duration of problems (Espie *et al*., 2018). The psychometric properties of the SCI are well supported in general population samples (Espie *et al*., 2018), and the internal reliability of the SCI scale in this sample was excellent (*α* = 0.90).

#### Obsessive compulsive disorder (OCD)

The *Obsessive Compulsive Inventory-Revised* (OCI-R; Foa *et al*., 2002) is an 18-item measure of six different clusters of obsessive compulsive disorder (OCD) symptoms. Participants indicate how distressed or bothered they have been by these symptoms in the past month using a five-point Likert scale ranging from 0 (‘Not at all’) to 4 (‘Extremely’). Total scores range from 0 to 72, and while initial work suggested a cut-off score of ⩾21 to identify possible diagnostic cases (Foa *et al*., 2002), subsequent studies have recommended higher cut-off scores including ⩾28 (Abramovitch *et al*., 2020) and ⩾36 (Williams *et al*., 2013). Cases of OCD were thus based on scores ⩾36. The psychometric properties of the OCI-R have been evidenced in clinical and non-clinical samples (Wootton *et al*., 2015), and the internal reliability of the OCI-R scores in this sample was excellent (*α* = 0.95).

#### AUD

AUD was assessed using the three-item *Alcohol Use Disorders Identification Test-Concise* (AUDIT-C; Bush *et al*., 1998). The AUDIT-C is brief version of the 10-item AUDIT scale developed by the WHO to assess hazardous drinking. Participants are asked three questions about their drinking behaviour over the past year with responses coded on a 0–4 Likert scale. Possible scores range from 0 to 12, and scores ⩾5 indicate a possible drinking problem. Scores of 5–7 indicate ‘increasing risk’, while scores ⩾8 indicate ‘high risk or possible dependence’ (UK Government, 2022). Scores ⩾8 have been found to maximise the specificity and sensitivity of detecting AUD in men and women (Khadjesari *et al*., 2017), and was therefore used in this study to classify participants as having AUD. The psychometric properties of the AUDIT-C have been supported in multiple studies (Jeong *et al*., 2017; Campbell and Maisto, 2018), and the internal reliability of the AUDIT-C scores in this sample was good (*α* = 0.76).

#### Psychosis

The *Psychosis Screening Questionnaire* (PSQ; Bebbington and Nayani, 1995) was used to measure the presence of different psychotic symptoms in the past year. The PSQ measures five symptoms of psychosis, including mania, thought insertion, paranoia, strange experiences and auditory/visual hallucinations. Participants are asked to indicate on a ‘Yes’ or ‘No’ basis if they have had each experience, and one or two follow-up questions are used to corroborate if the experience was reflective of a psychotic symptom. Symptom endorsement is based on affirmative responses to the initial probe and follow-up questions. Although the PSQ has been widely used to screen for psychosis in multiple populations and has been shown to possess excellent psychometric properties (Heuvelman *et al*., 2018), it does not have a threshold score to indicate the presence of a psychotic disorder. However, in an assessment of the validity of psychosis screening questionnaires to detect clinically relevant psychotic disorders, Kelleher *et al*. (2011) showed that items measuring auditory and visual hallucinations have exceptional predictive power for identifying interview-verified psychosis disorders. Thus, in this study, we classified participants as having a psychosis disorder if they endorsed the final two-part item on the PSQ measuring auditory and visual hallucinations: ‘*Over the past year, have there been times when you heard or saw things that other people couldn't?*’ and ‘*Did you at any time hear voices saying quite a few words or sentences when there was no one around that might account for it?*’. The internal reliability of the PSQ scores in this sample was *α* = 0.64. While this is somewhat lower than desirable, the small number of items and binary response format is likely to have contributed to the low-scale *α*.

#### Personality disorders

Four personality disorders were assessed using the same items and diagnostic rules used in the UK's Psychiatric Morbidity among Adults Living in Private Households Survey (Singleton *et al*., 2003). These items were derived from *The Structured Clinical Interview for DSM-IV Axis II Disorders* (First *et al*., 1997). Participants were given the following instruction: ‘*These questions are about the kind of person you generally are, that is, how you have usually felt or behaved over the past several years*’, and asked to answer all questions on a ‘Yes’ or ‘No’ basis. Avoidant personality disorder was assessed using seven items (*α* = 0.79), and diagnostic criteria were met if six of seven symptoms were endorsed. Fifteen items were used to assess the nine symptoms of borderline personality disorder (*α* = 0.89), and diagnostic criteria were met if seven of nine symptoms were endorsed. Histrionic personality disorder was assessed using six items (*α* = 0.66), and diagnostic criteria were met if all six symptoms were endorsed. Finally, schizoid personality disorder was assessed using seven items (*α* = 0.58), and diagnostic criteria were met if six of seven items were endorsed.

#### Attempted suicide

Attempted suicide was measured using one item taken from the 2014 English Adult Psychiatric Morbidity Survey (McManus *et al*., 2016). Participants were asked, ‘*Have you ever made an attempt to take your own life?*’ and were asked to answer using a ‘Yes’ or ‘No’ response format.

### Data analysis

The prevalence rates of the 12 mental health disorders were estimated along with the proportion of people meeting criteria for any of these disorders. Associations between the different disorders were assessed using tetrachoric correlations. Next, binary logistic regression analysis was used to identify the sociodemographic variables uniquely associated with meeting criteria for any mental health disorder. The predictor variables in the model were the sociodemographic variables listed in Table 1, and the magnitude of the associations is reported as adjusted odds ratios (AORs). Next, the association between meeting criteria for any mental health disorder and a lifetime history of attempted suicide was assessed using a Pearson *χ*^2^ test of association, and the magnitude of this association is reported as an unadjusted odds ratio (OR). Unadjusted and adjusted associations between meeting criteria for each mental health disorder and having attempted suicide were assessed using *χ*^2^ tests of association and binary logistic regression analysis, respectively. Finally, adjusted population attributable risk fractions (PAFs) were calculated to determine the proportion of attempted suicides that were uniquely related to each mental health disorder, while adjusting for the shared variance among all disorders. PAFs indicate the proportion of a given outcome in the population (i.e. attempted suicide) that would be prevented if a given risk factor (i.e. a particular mental health disorder) could be eliminated, assuming a causal relationship between the risk factor and the outcome. PAFs are a useful way of calculating the unique effect of a given risk factor because they consider the prevalence of that risk factor in the population. Thus, a mental health disorder with a low prevalence and a high association with attempted suicide may have less of an effect than a disorder with a high prevalence and a lower association with attempted suicide.

## Results

**Fig. 1.**
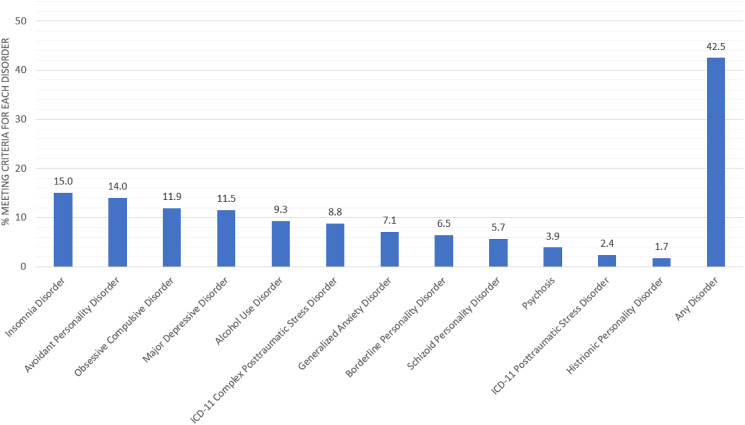
Prevalence estimates of each mental health disorder and of any mental health disorder.

The following proportion of adults screened positive for each disorder: insomnia disorder (15.0%, 95% CI 12.9–17.2%), avoidant personality disorder (14.0%, 95% CI 11.9–16.0%), OCD (11.9%, 95% CI 10.0–13.8%), MDD (11.5%, 95% CI 9.6–13.4%), AUD (9.3%, 95% CI 7.6–11.0%), ICD-11 CPTSD (8.8%, 95% CI 7.2–10.5%), GAD (7.1%, 95% CI 5.6–8.6%), borderline personality disorder (6.5%, 95% CI 5.0–7.9%), schizoid personality disorder (5.7%, 95% CI 4.3–7.0%), psychosis (3.9%, 95% CI 2.7–5.0%), ICD-11 PTSD (2.4%, 95% CI 1.5–3.3%) and histrionic personality disorder (1.7%, 95% CI 0.9–2.5%). Overall, 42.5% (95% CI 39.6–45.4%) screened positive for any of the above mental health disorders (see Fig. 1).

**Fig. 2.**
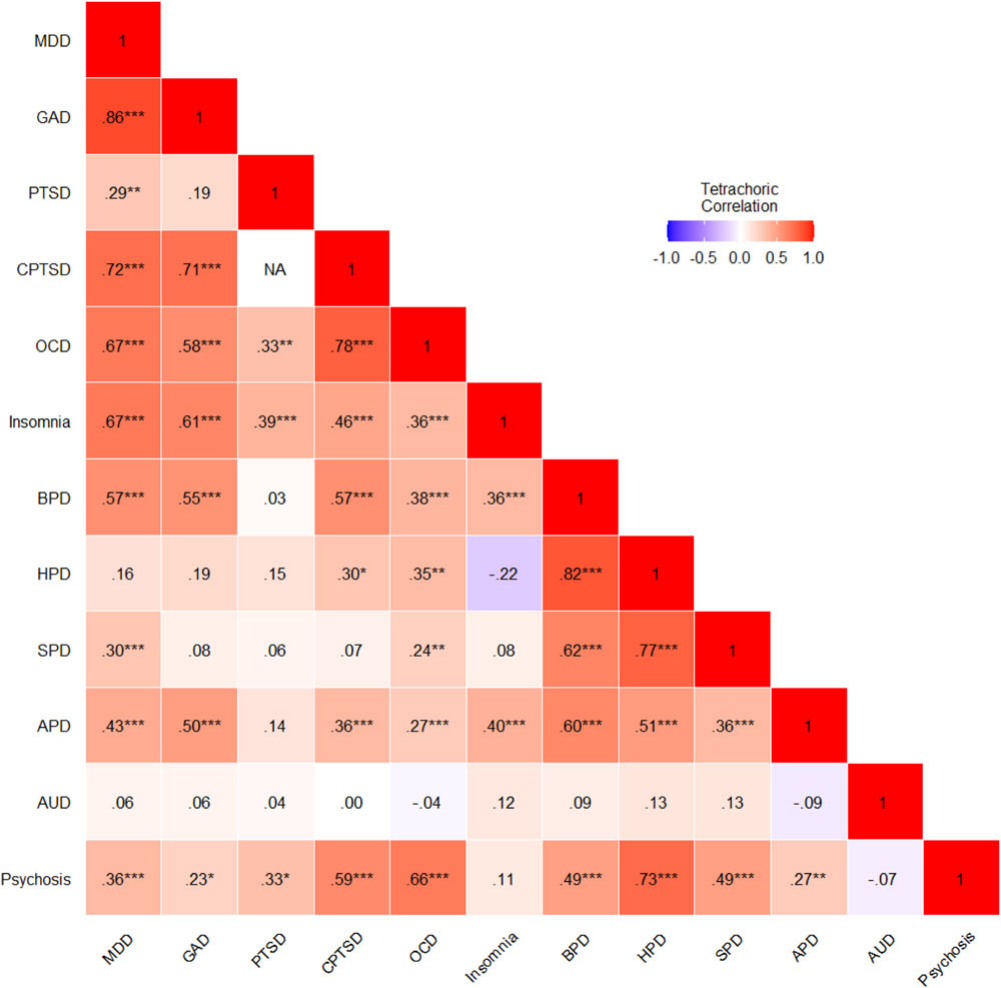
Tetrachoric correlations among all mental health disorders. MDD, major depressive disorder; GAD, generalised anxiety disorder; PTSD, ICD-11 posttraumatic stress disorder; CPTSD, ICD-11 complex PTSD; OCD, obsessive compulsive disorder; BPD, borderline personality disorder; HPD, histrionic PD; SPD, schizoid PD; APD, avoidant PD; AUD, alcohol use disorder.

The correlations between the 12 disorders are presented in Fig. 2. Of the 65 correlations^1^, 61 were positive and 42 were statistically significant (*p* < 0.05). AUD was the only disorder uncorrelated with any other disorder.

Table 2 presents the results of the binary logistic regression model of meeting criteria for any mental health disorder. The model was statistically significant (*χ*^2^ (34, *n* = 1107) = 139.91, *p* < 0.001), and several variables were uniquely associated with meeting criteria for any mental health disorder. Compared to those aged 55 years and older, those aged 18–24 (AOR = 8.00), 25–34 (AOR = 3.15), 35–44 (AOR = 2.37) and 45–54 (AOR = 1.69) were significantly more likely to meet criteria for having a disorder. Compared to those who were in full-time employment, full-time students were significantly less likely to meet criteria for having a disorder (AOR = 0.40). Shift-workers were significantly more likely than non-shift workers to meet criteria for having a disorder (AOR = 1.70). Trauma-exposed persons were significantly more likely than non-trauma-exposed persons to meet criteria for having a disorder (AOR = 2.15).

**Table 2.** Associations between sociodemographic variables and meeting criteria for any mental health disorder

	*B*	s.e.	Wald	*p*	AOR (95% CI)		
Females	−0.17	0.14	1.48	0.224	0.84	0.64	1.11
Age (55+)							
**18–24**	**2**.**08**	**0**.**35**	**35**.**93**	**<0**.**001**	**8**.**00**	**4**.**05**	**15**.**79**
**25–34**	**1**.**15**	**0**.**25**	**20**.**74**	**<0**.**001**	**3**.**15**	**1**.**92**	**5**.**17**
**35–44**	**0**.**86**	**0**.**24**	**13**.**28**	**<0**.**001**	**2**.**37**	**1**.**49**	**3**.**77**
**45–54**	**0**.**53**	**0**.**23**	**5**.**10**	**0**.**024**	**1**.**69**	**1**.**07**	**2**.**68**
Born in Ireland	0.08	0.25	0.10	0.747	1.08	0.67	1.76
Irish ethnicity	−0.40	0.27	2.18	0.140	0.67	0.39	1.14
Living area (rural)							
City	−0.06	0.20	0.09	0.764	0.94	0.64	1.38
Suburb	0.15	0.20	0.55	0.460	1.16	0.79	1.70
Town	−0.08	0.18	0.17	0.679	0.93	0.65	1.33
Education (finished secondary school)							
Undergraduate degree	−0.24	0.20	1.48	0.224	0.79	0.54	1.16
Postgraduate degree	−0.18	0.22	0.66	0.417	0.84	0.54	1.29
Other qualification	−0.23	0.20	1.41	0.236	0.79	0.54	1.16
Did not finish secondary school	0.16	0.30	0.27	0.606	1.17	0.65	2.11
Religious	0.19	0.18	1.19	0.275	1.21	0.86	1.71
Relationship status (in a committed relationship)							
Single	−0.08	0.20	0.16	0.691	0.93	0.63	1.36
Separated/divorced	0.26	0.32	0.70	0.402	1.30	0.70	2.42
Widowed	−0.43	0.51	0.71	0.399	0.65	0.24	1.76
Living alone	0.33	0.23	2.10	0.148	1.39	0.89	2.17
Children (no)							
Yes, living with me	0.21	0.18	1.35	0.245	1.23	0.87	1.75
Yes, not living with me	0.03	0.25	0.01	0.923	1.03	0.62	1.69
Employment status (full-time employed)							
Part-time employed	−0.05	0.22	0.04	0.836	0.96	0.62	1.47
Temporary working arrangement	−0.42	0.47	0.82	0.366	0.66	0.26	1.64
Retired	−0.29	0.27	1.16	0.282	0.75	0.45	1.27
Unemployed	0.10	0.25	0.17	0.684	1.11	0.68	1.82
Temporarily unemployed COVID-19	−0.40	0.30	1.80	0.180	0.67	0.37	1.20
**Full-time student**	**−0**.**91**	**0**.**39**	**5**.**51**	**0**.**019**	**0**.**40**	**0**.**19**	**0**.**86**
Income level (€50 000 or more)							
€0–19 999	0.44	0.25	3.02	0.082	1.55	0.95	2.55
€20 000–29 999	0.31	0.24	1.75	0.187	1.37	0.86	2.17
€30 000–39 999	0.13	0.24	0.33	0.568	1.14	0.72	1.81
€40 000–49 999	0.40	0.25	2.60	0.107	1.49	0.92	2.43
**Shift worker**	**0**.**53**	**0**.**20**	**7**.**10**	**0**.**008**	**1**.**70**	**1**.**15**	**2**.**52**
Physical health condition	0.14	0.15	0.85	0.357	1.15	0.85	1.56
**Trauma exposed**	**0**.**77**	**0**.**15**	**26**.**01**	**<0**.**001**	**2**.**15**	**1**.**60**	**2**.**89**

*Note:* Statistically significant associations are in bold; the reference category for ordinal-level predictor variables is in parenthesis.

Lifetime history of attempted suicide was reported by 11.1% (95% CI 9.2–12.9%), and those who met criteria for any mental health disorder were significantly more likely than those without a disorder to have attempted suicide (20.0 *v.* 4.6%, *χ*^2^ (1, *n* = 1101) = 65.38, *p* < 0.001, OR = 5.26, 95% CI 3.40–8.13). The unadjusted and adjusted associations between meeting criteria for each mental health disorder and having attempted suicide are reported in Table 3. With the exceptions of AUD and ICD-11 PTSD, meeting criteria for each disorder was bivariately associated with having attempted suicide. The binary logistic regression model, with all mental health disorders entered as predictor variables of attempted suicide, was statistically significant (*χ*^2^ (12, *n* = 1101) = 128.63, *p* < 0.001). Five disorders were uniquely associated with having attempted suicide: avoidant personality disorder (AOR = 1.68), insomnia disorder (AOR = 1.94), ICD-11 CPTSD (AOR = 2.40), psychosis (AOR = 3.01) and borderline personality disorder (AOR = 3.05).

**Table 3.** Unadjusted and adjusted odds ratios of the associations between each mental health disorder and lifetime history of attempted suicide

	OR (95% CI)			AOR (95% CI)		
Insomnia disorder	**3**.**40**	**2**.**23**	**5**.**18**	**1**.**94**	**1**.**15**	**3**.**27**
Avoidant personality disorder	**3**.**74**	**2**.**44**	**5**.**72**	**1**.**68**	**1**.**00**	**2**.**84**
Obsessive compulsive disorder	**4**.**67**	**3**.**03**	**7**.**29**	1.64	0.90	2.96
Major depressive disorder	**4**.**58**	**2**.**95**	**7**.**12**	0.97	0.47	1.91
Alcohol use disorder	0.86	0.45	1.70	0.78	0.37	1.66
ICD-11 complex posttraumatic stress disorder	**7**.**18**	**4**.**51**	**11**.**45**	**2**.**40**	**1**.**25**	**4**.**61**
Generalised anxiety disorder	**5**.**53**	**3**.**33**	**9**.**21**	1.61	0.78	3.30
Borderline personality disorder	**8**.**57**	**5**.**12**	**14**.**34**	**3**.**05**	**1**.**55**	**5**.**98**
Schizoid personality disorder	**3**.**28**	**1**.**82**	**5**.**93**	1.47	0.66	3.28
Psychosis	**8**.**15**	**4**.**33**	**15**.**35**	**3**.**01**	**1**.**37**	**6**.**61**
ICD-11 posttraumatic stress disorder	2.48	0.98	6.30	1.86	0.65	5.33
Histrionic personality disorder	**7**.**72**	**3**.**07**	**19**.**39**	1.21	0.35	4.21
Any mental health disorder	**5**.**26**	**3**.**40**	**8**.**13**	–	–	–

*Note*: Statistically significant (*p* < 0.05) effects are highlighted in bold.

**Fig. 3.**
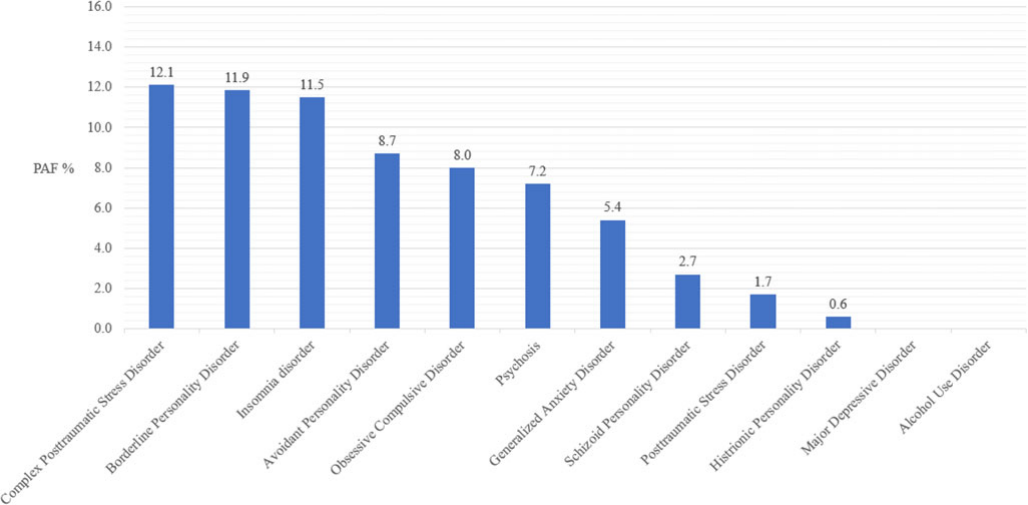
Adjusted population attributable risk fractions for attempted suicide based on the unique effect of each mental health disorder.

The adjusted PAFs for attempted suicide are presented in Fig. 3. The highest PAFs were for ICD-11 CPTSD (12.1%), borderline personality disorders (11.9%) and insomnia disorder (11.5%).

## Discussion

This study was carried out to estimate the prevalence rates of multiple mental health disorders in the general adult population of Ireland, to identify who in the population was most likely to have a mental health disorder, and to determine the unique associations between each disorder and attempted suicide. Prevalence estimates for individual disorders ranged from 1.7% (histrionic personality disorder) to 15.0% (insomnia disorder). Four disorders – insomnia disorder, avoidant personality disorder, OCD and MDD – had a prevalence above 10%, and three disorders – histrionic personality disorder, psychosis and ICD-11 PTSD – had a prevalence below 5%. These estimates are broadly in-line with international figures. For example, at least 10% of the general population in multiple nations meet criteria for insomnia disorder (Roth, 2007); 8.8% of adults in Europe meet criteria for AUD (WHO, 2018); and, across 52 nations, 5.8% of people in the general population report symptomatic hallucinations – consistent with our measurement of psychosis (Nuevo *et al*., 2012).

The overall figure of 42.5% of adults in Ireland meeting criteria for any one of the 12 mental health disorders is consistent with findings from the Dunedin birth cohort data from New Zealand, where 44–50% of people met criteria for one of 11 mental health disorders at various points during adulthood (Caspi *et al*., 2020). Moreover, this figure is also in-line with those nations located in the upper quartile of the distribution from the World Mental Health Survey (Kessler *et al*., 2007). More specifically, nations with a similar proportion of their adult population meeting criteria for a mental health disorder include France (38%), Columbia (39%), New Zealand (39%) and the USA (47%). Notably, neighbouring European countries such as the Netherlands (31%), Belgium (29%), Germany (25%), Spain (19%) and Italy (18%) were found to have a smaller proportion of their adult population meeting criteria for a disorder. Based on how many people in Ireland appear to be affected by mental health problems, mental health care should be approached with the same zeal as other common causes of disability in Ireland, such as ischaemic heart disease, chronic pain and Alzheimer's and other dementias (WHO, 2019). This includes careful and structured monitoring and assessment of risk factors, rapid treatment and regular follow-up, and education and awareness raising.

Consistent with a well-established literature on the comorbidity of mental health disorders (Plana-Ripoll *et al*., 2019), we found strong, positive associations between the various disorders. Here, two findings are worth highlighting. First, particularly strong associations were found between disorders that can be categorised as ‘internalising’ disorders (indicated in Fig. 2 by the clustering of deep red towards the upper-left quadrant). Recently, Watson *et al*. (2022, p. 26) proposed a dimension of ‘emotional dysfunction’ to explain the co-occurrence of these disorders, noting that these disorders ‘*share genetic diatheses, environmental risk factors, cognitive and affective difficulties, neural substrates and biomarkers, childhood temperamental antecedents, and treatment response*’. Second, and running counter to what is expected given the known common occurrence of AUD with many psychiatric disorders (Castillo-Carniglia *et al*., 2019), AUD was uncorrelated with all other disorders. Multiple sensitivity checks were conducted to probe the source of this unusual result including setting different cut-off scores on the AUDIT-C and performing correlation tests using continuous scoring methods. All produced the same null effect. It is difficult to say, therefore, why AUD was uncorrelated with all other disorders. It may be a measurement/methodological issue or, perhaps, a cultural issue specific to the Irish population. Only future research will illuminate the reason for this result.

Sociodemographic factors independently associated with higher odds of meeting criteria for a mental health disorder included younger age, being a shift worker and trauma exposure, while being a student (compared to being in full-time employment) was independently associated with lower odds of having a disorder. While there is much evidence that mental health disorders are particularly common among university students (Ibrahim *et al*., 2013; Auerbach *et al*., 2016, 2018), our findings suggest that this effect is largely driven by age, rather than being in university. In fact, our results suggest that being in university is associated with a *lower* risk of mental health problems. While the mechanisms underlying this finding are not clear, results may be attributable to readily available mental health services (e.g. student counselling services) and greater socialisation (Ma *et al*., 2020), as characteristics of a university setting.

That said, individuals aged 18–24 were still eight times more likely than those aged 55 and older to have a mental health disorder. This finding is consistent with the idea that the three first decades of life are the most critical for the onset of mental health disorders (Kessler *et al*., 2007; Jones, 2013), and while we did not sample individuals under the age of 18, it points to the importance of increasing mental health screening, prevention and intervention services for younger people. Increasing access to mental health services for children and younger adults is particularly important when we consider the growing number of referrals for child and adolescent mental health services in Ireland (McNicholas *et al*., 2021), and that unlike other aetiologies of disability, 63% of people with a mental disorder report onset before the age of 25 (Solmi *et al*., 2022).

In addition, we found that shift workers (i.e. those that work outside the hours of 7am and 6pm) were 70% more likely than non-shift workers to meet criteria for a mental health disorder. The pernicious physical and mental health effects of shift work are well documented, and are believed to be driven, in part, by disruptions to circadian systems in the body (Vogel *et al*., 2012; McGowan and Coogan, 2013; Brown *et al*., 2020). Notably, in 2019 the International Agency for Research on Cancer concluded that ‘night shift work’ is probably carcinogenic to humans (IRAC, 2019). With approximately 15% of the Irish workforce engaged in shift work (Health and Safety Authority, 2021), universal public (mental and physical) health interventions targeting shift workers are likely to be particularly beneficial. Furthermore, this finding highlights the need for companies employing shift workers to consider additional statutory duties to ensure the health and safety of their employees.

The link between trauma exposure and mental health disorders is also well-established, with evidence indicating that trauma exposure precedes the onset of mental health problems (Read *et al*., 2001; McKay *et al*., 2021). We found that 70% of adults in Ireland had experienced at least one traumatic event in their lifetime, and this matches global assessments of the proportion of people who have experienced a traumatic life event (Benjet *et al*., 2016). This suggests that even a small reduction in the occurrence of trauma in society could result in positive population-level mental health effects and highlights the importance of supporting services and organisations providing critical care to individuals in the wake of a traumatic life event.

Consistent with recent estimates from the Irish adult population (Hyland *et al*., 2022), we found that approximately one-in-ten people reported a lifetime history of attempted suicide. Those with a mental health disorder were more than five times as likely to have attempted suicide than those without a mental health disorder. This figure is consistent with data-linkage studies indicating that those with a mental disorder are seven-and-a-half times more likely to die by suicide than those without a disorder (Too *et al*., 2019).

Five mental health disorders were independently associated with having attempted suicide: BPD, psychosis, ICD-11 CPTSD, insomnia disorder and avoidant personality disorders. Given the variation in the prevalence of these disorders, the PAFs allowed us to understand the unique contribution of each disorder to attempted suicide. For example, although insomnia disorder was less strongly associated with attempted suicide than psychosis (AOR = 1.94 *v*. 3.01), the higher prevalence of insomnia disorder relative to psychosis (15.0 *v*. 3.9%) means that the elimination of insomnia disorder would be associated with a larger decline in attempted suicides compared to the elimination of psychosis (11.5 *v*. 7.2%). Considering the logistic regression and PAF analyses together, these findings indicate, for example, that for any given individual the odds of an attempted suicide are considerably higher if that person has a psychotic disorder rather than insomnia disorder; however, at the population level, the impact of insomnia disorder on rates of attempted suicide is greater precisely because of the much higher prevalence of insomnia disorder. This would indicate that, for example, if a patient was to present with both psychotic and insomnia symptoms in a clinical setting, the treatment of the psychotic symptoms would take precedence (if both could not be treated concurrently), given the increased odds ratio of psychosis compared to insomnia disorder. However, if funding was being used to administer a widespread intervention to reduce the risk of attempted suicide among the general population, it may be more prudent to target the reduction/prevention of symptoms of insomnia disorder as this would, theoretically, lead to a larger reduction in overall attempted suicides, given the increased PAF for insomnia disorder compared to psychosis.

The finding that the PAF for MDD was zero may strike some readers as unusual given both the prevalence of MDD in this sample (11.5%), and the well-established links between MDD and suicide-related phenomena (Cai *et al*., 2021). This finding was almost certainly due to the high degree of association between MDD and most other disorders, particularly those reflecting emotional dysfunction (Watson *et al*., 2022). One should not conclude therefore that MDD is unrelated to a history of attempted suicide – indeed, the bivariate association was very strong (OR = 4.58) – but rather that after adjusting for the myriad comorbidities associated with MDD, MDD does not have a *unique* effect on attempted suicide. From a clinical perspective, MDD is likely to be crucially related to suicide-related phenomena but among MDD patients at risk of suicide there are likely to be many mental health comorbidities that are contributing to the risk of suicide. Alleviation of MDD and comorbid disorders is likely to be necessary to lower the risk of suicide.

Despite the high prevalence of mental health disorders in Ireland, only 5.1% of Ireland's total government health expenditure is allocated to mental health (Mental Health Reform Ireland, 2021). This figure is well below the 12% expenditure recommended by the WHO and the 10% expenditure targeted by Ireland's health care reform, Sláintecare. Consonant with such a small proportion of total health spending on mental health care is a shortage of human resources for mental health. Ireland has 18 psychiatrists per 100 000 of the population – lower than Switzerland (52), New Zealand (29), Germany (27) and France (23) (Eurostat, 2020; WHO, 2022) – and is currently experiencing a 30% vacancy in permanent consultant psychiatry posts (Irish Hospital Consultants Association [IHCA], 2020). Similarly, Ireland only has 22 acute mental health beds per 100 000 population, lagging significantly behind the EU average of 70 per 100 000 (IHCA, 2020). Increasing the resources available for mental health services, however, must be met with concurrent efforts to increase engagement with and access to acceptable and comprehensive forms of care for individuals and their families.

There are several limitations that ought to be considered. First, our results were derived from a non-probability sample, and while efforts were taken to ensure its representativeness, the findings may not generalise to the entire adult population of Ireland. Second, prevalence rates were obtained using self-report measures, which are known to yield higher rates than diagnostic interviews. While we tried to offset this potential inflation of prevalence rates by using the strictest possible criteria for probable diagnosis, our findings may represent an over-estimation of the true population prevalence of mental health disorders in Ireland. That said, our sample also lacked representation from subsets of the population known to have high rates of mental disorders including prisoners (Kennedy *et al*., 2005; Fazel and Seewald, 2012), the homeless (Prinsloo *et al*., 2012) and those who are institutionalised. Third, the data are cross-sectional, so no causal inferences can be made. Fourth, this study was carried out during a period of strict national public health measures to control the spread of COVID-19 possibly influencing these findings. However, we believe it is unlikely that the current estimates were markedly affected by the context of COVID-19 as there is evidence that the proportion of Irish adults screening positive for depression and anxiety did not change from before and after the outbreak of COVID-19 (Daly *et al*., 2021), nor did symptoms or rates of depression, anxiety or COVID-19-related PTSD change substantially during the first 12 months of the pandemic (Hyland *et al*., 2021c).

Despite these limitations, this study provides the most comprehensive assessment of the prevalence and co-occurrence of mental health disorders among adults living in Ireland. Universal public mental health strategies targeting young adults, those employed in occupations that involve shift work, and those with a history of trauma are particularly needed to rein in the economic and human impact of mental health disorders. Effective treatments for relatively common disorders such as ICD-11 CPTSD, BPD and insomnia disorder are also required to achieve substantial reductions in attempted suicide.

## Data

All data used in this study is freely available at https://osf.io/c57fp/

## Footnotes

1 There are 65 rather than 66 correlations because PTSD and CPTSD are independent by the nature of their diagnostic rules.
